# Proximal Peril From a Distal Malignancy: Impending Colonic Perforation in Obstructing Sigmoid Adenocarcinoma

**DOI:** 10.7759/cureus.111799

**Published:** 2026-06-30

**Authors:** Minesh Sindhal, Priyanka Aanandaka, Mohith VS, Divyeshkumar N Parmar, Anand Kumar

**Affiliations:** 1 Surgery, All India Institute of Medical Sciences, Rajkot, Rajkot, IND; 2 General Surgery, All India Institute of Medical Sciences, Rajkot, Rajkot, IND

**Keywords:** cecal perforation, closed-loop obstruction, colorectal cancer, emergency colectomy, malignant large bowel obstruction, sigmoid adenocarcinoma

## Abstract

Sigmoid colon adenocarcinoma is a common cause of large bowel obstruction in elderly patients. However, progression to impending proximal colonic perforation due to distal malignant obstruction represents a rare and catastrophic complication associated with high morbidity and mortality. A 74-year-old female presented with acute constipation of five days’ duration, progressive abdominal distension, nausea, vomiting, and one episode of fever. Contrast-enhanced computed tomography demonstrated diffuse dilatation of the large bowel, multiple air-fluid levels, and an irregular circumferential wall thickening of the sigmoid colon over a 6-cm segment, causing distal luminal narrowing with marked upstream dilatation and fecalization of the ileum, radiologically consistent with malignant obstruction. Emergency exploratory laparotomy revealed an obstructing sigmoid mass with gross proximal colonic distension and features of impending perforation involving the cecum and hepatic flexure. The patient underwent sigmoid resection with primary anastomosis, extended right hemicolectomy with end-to-side ileo-transverse anastomosis, omentectomy, and thorough peritoneal lavage. Histopathology confirmed a well-differentiated (Grade 1) adenocarcinoma of the sigmoid colon. Distal sigmoid malignancy can precipitate closed-loop large bowel obstruction with progressive proximal colonic dilatation, culminating in impending perforation at remote colonic segments. Early recognition and timely definitive surgical intervention are critical to prevent fatal outcomes.

## Introduction

Colorectal carcinoma is among the leading causes of cancer-related morbidity and mortality worldwide [1]. Obstruction occurs in approximately 8-29% of colorectal cancer cases and is more commonly associated with left-sided tumors due to the narrower lumen and firmer fecal consistency [2,3].

In the presence of a competent ileocecal valve, distal obstruction produces a closed-loop phenomenon. Progressive colonic distension increases intraluminal pressure, compromises mural perfusion, and may lead to ischemia and perforation [2,4]. According to Laplace’s law, wall tension is proportional to luminal radius, making the cecum the most vulnerable segment [2,5]. However, involvement of additional proximal segments, such as the hepatic flexure, reflects severe intraluminal hypertension and advanced pathophysiological compromise.

We report a rare case of sigmoid adenocarcinoma presenting with impending perforation involving both the cecum and hepatic flexure.

## Case presentation

A 74-year-old female presented to the emergency department with a five-day history of absolute constipation, progressive abdominal distension, and discomfort. There was no prior history of gastrointestinal malignancy, inflammatory bowel disease, or abdominal surgery. No significant family history of colorectal cancer was reported.

Clinical examination

On physical examination, the patient was hemodynamically stable. The abdomen was distended and non-tender. Bowel sounds were sluggish, and there were no signs of generalized peritonitis. Digital rectal examination revealed the rectum to be dilated and empty of stool. Table 1 presents the laboratory investigation.

**Table 1:** Laboratory investigations

Parameter	Day 1	Day 11	Reference Range
Hb (g/dL)	9.6	-	12–17
WBC (cells/µL)	12000	-	4000–11000
Platelets (×10³/µL)	390	-	150–450
CEA (ng/mL)	-	1.22	0–3
CA 19-9 (U/mL)	-	10.13	0–37

Contrast-enhanced computed tomography (CECT) of the abdomen and pelvis demonstrated gross dilatation of large bowel loops involving the ascending, transverse, and descending colon, with a maximum diameter of 7.1 cm and a dilated cecum measuring approximately 11 cm (Figure 1). Multiple air-fluid levels, irregular circumferential wall thickening measuring approximately 7.2 mm in the sigmoid colon over a segment of 6 cm, and mild luminal narrowing in the sigmoid colon with marked upstream dilatation with fecalization of the ileum were also observed (Figure 2). These findings were consistent with malignant distal large bowel obstruction with significant proximal colonic dilatation.

**Figure 1:**
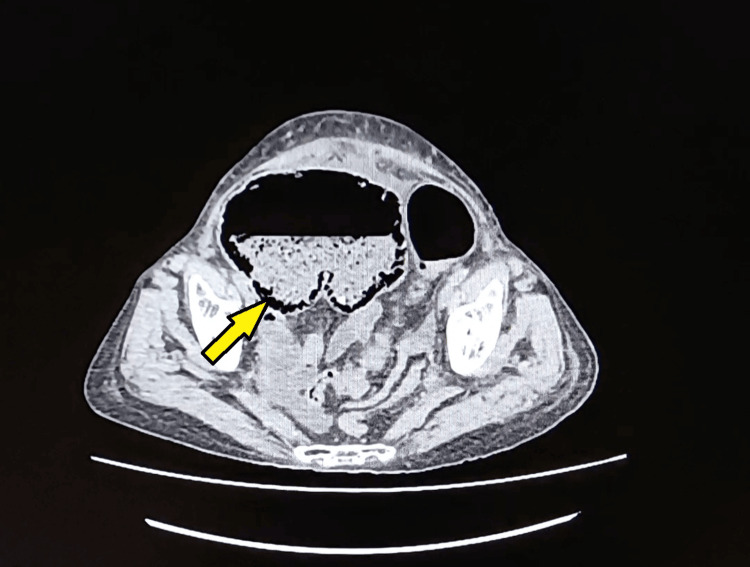
Axial section of CECT abdomen showing a dilated cecum (yellow arrow) measuring approximately 11 cm

**Figure 2:**
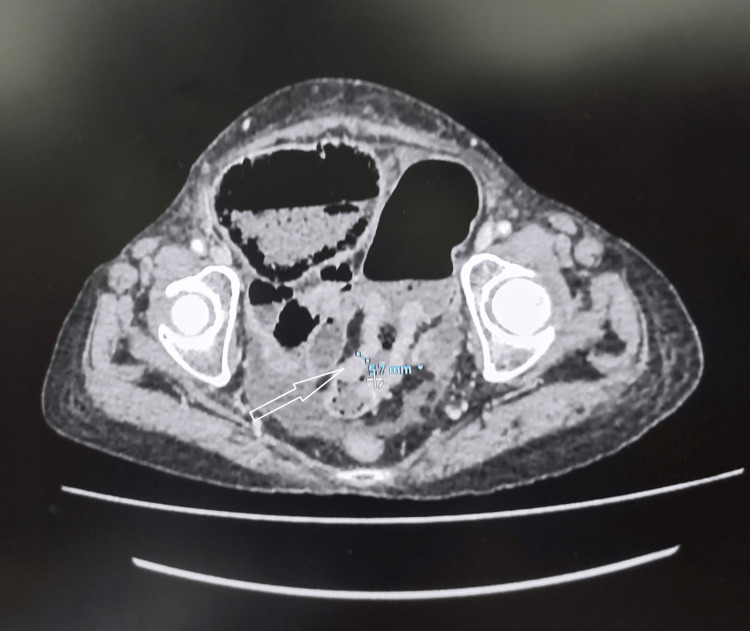
Axial section of CECT abdomen showing luminal narrowing (white arrow) of the sigmoid colon (obstruction)

Diagnosis

The diagnosis is malignant large bowel obstruction secondary to sigmoid colon carcinoma, with impending proximal colonic perforation.

Therapeutic intervention

The patient underwent emergency exploratory laparotomy. Intraoperative findings included an obstructing circumferential mass lesion at the junction of the descending colon and sigmoid colon, measuring 3x5 cm, involving the entire bowel wall, causing luminal narrowing and complete large bowel obstruction, with a dilated proximal colon with fecal loading and a collapsed distal sigmoid colon and rectum. Serosal thickening is suggestive of transmural involvement along the mass. Dense adhesions are present between the mass and the left-sided broad, round ligament. A palpable enlarged lymph node was found along the left para-aortic region. Moderate ascites with blood-tinged, straw-colored, odorless exudates is present. A serosal tear is present along the large bowel. There is one lesion at the proximal cecum and two lesions at the transverse colon, with impending perforation and marked dilatation of the cecum, ascending colon, and transverse colon, with bowel edema and dusky color, suggesting compromised vascularity. The rest of the examined structures appeared normal. There was no gross evidence of distant metastasis.

Resection of the sigmoid mass with primary descending colon-sigmoid anastomosis, extended right hemicolectomy, omentectomy, and end-to-side ileo-transverse anastomosis and thorough peritoneal lavage were done (Figures 3, 4).

**Figure 3:**
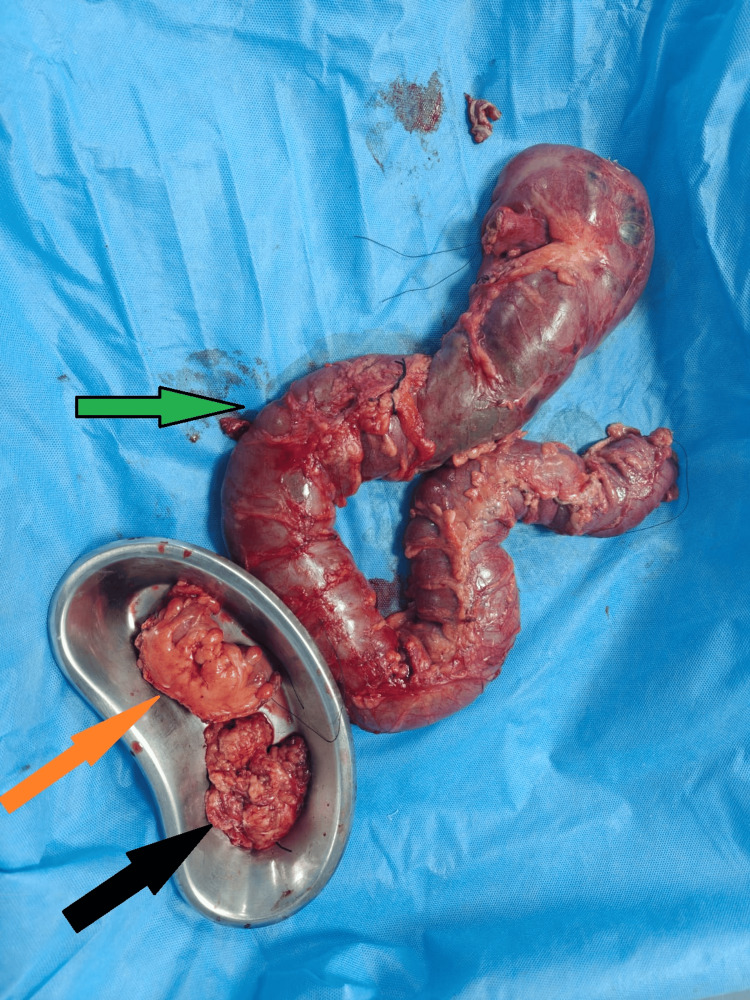
Specimen of resected sigmoid mass (orange arrow), extended right hemicolectomy (green arrow), omentectomy (black arrow)

**Figure 4:**
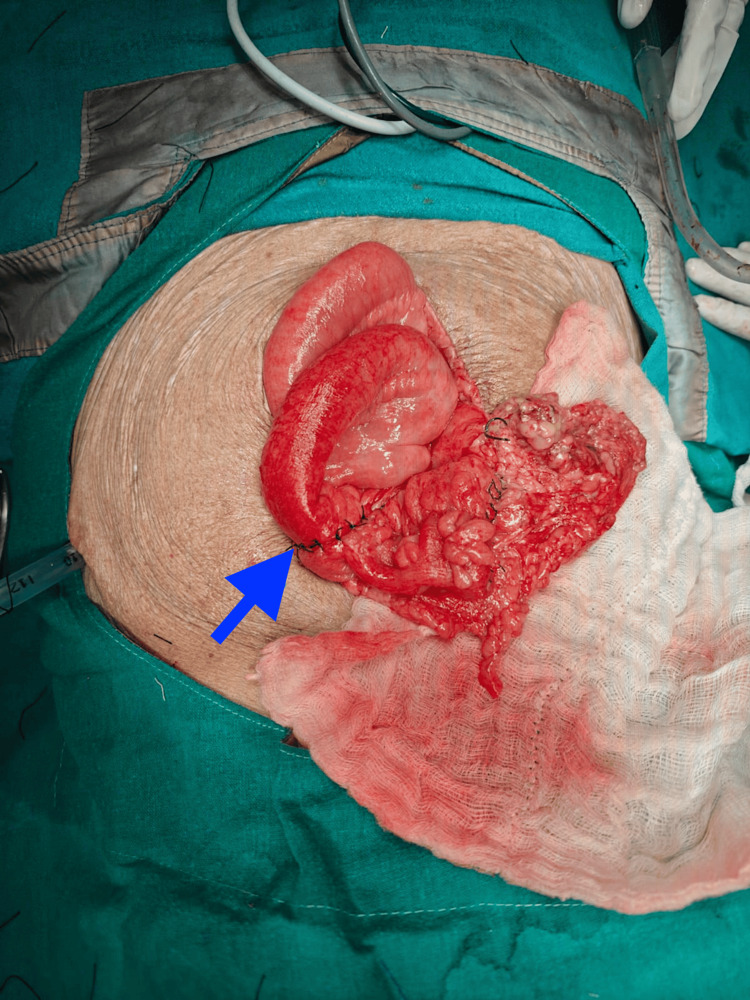
Image showing end-to-side ileo-transverse anastomosis (blue arrow)

Histopathological findings

Sections studied from the growth revealed an invasive malignant tumor with extensive areas of necrosis. Atypical mitosis was noted. The tumor invades the muscularis propria and subserosal fat. The serosa is free of tumor, with a 1 mm clearance. Lymphovascular emboli and perineural invasion are not seen. Both resection margins are free of tumor. Adjacent mucosa was unremarkable. No lymph nodes were identified along the specimen. Well-differentiated adenocarcinoma of the sigmoid colon (Grade 1). All margins are negative for invasive carcinoma.

Outcome and follow-up

The postoperative course was uneventful. The patient was referred to medical oncology for further staging workup and adjuvant therapy planning as per oncological guidelines [6].

## Discussion

Large bowel obstruction due to left-sided colorectal cancer is a well-recognized surgical emergency [2,3]. In the presence of a competent ileocecal valve, obstruction results in a closed-loop system. Progressive intraluminal pressure may exceed capillary perfusion pressure, leading to ischemia and transmural necrosis [2].

Cecal diameters exceeding 12 cm are associated with increased risk of perforation due to maximal wall tension [4]. Although the cecum is classically the most vulnerable site, extreme pressure dynamics may involve additional segments such as the hepatic flexure, as seen in this case.

Emergency resection remains the definitive treatment. The choice between primary anastomosis and staged procedures depends on patient stability, contamination risk, and bowel viability [2,4,6]. Early CT imaging is crucial in identifying impending perforation and guiding timely surgical management.

## Conclusions

Obstructing sigmoid adenocarcinoma may rarely present with impending proximal colonic perforation due to closed-loop large bowel obstruction. Recognition of this pathophysiological progression and urgent definitive surgical management are essential to prevent fatal outcomes. This case highlights the need for heightened clinical suspicion and prompt intervention in elderly patients presenting with acute constipation and abdominal distension.
